# Lessons and Perspectives from a 25-Year Bioelectromagnetics Research Program

**DOI:** 10.3390/ijerph13100950

**Published:** 2016-09-23

**Authors:** Andrew W. Wood, Alireza Lajevardipour, Robert L. McIntosh

**Affiliations:** 1 School of Health Sciences, Swinburne University of Technology, Melbourne, VIC 3122, Australia; alajevardipour@swin.edu.au (A.L.); Robert.L.McIntosh@team.telstra.com (R.L.M.); 2 Chief Technology Office, Telstra Corporation, Melbourne, VIC 3122, Australia

**Keywords:** electromagnetic fields, radiofrequency fields, magnetic fields, tissue electric properties

## Abstract

The question of whether electromagnetic fields from electric power or telecommunications systems can be linked unequivocally to health detriments has occupied scientific research endeavors for nearly half a century. For 25 years, the bioelectromagnetic research group at Swinburne University in Melbourne, Australia, has pursued a series of investigations with relevant endpoints, such as neurophysiological and neuropsychological effects, cell calcium level changes, proliferation, and genotoxic effects. Most have shown no significant changes due to fields, however, in some pilot studies significant changes were revealed, but in most cases these were not replicated in follow-up studies. This highlights a feature of this research area, generally; the unambiguous identification of small changes in noisy data where the understanding of possible interaction mechanisms is lacking. On the other hand, mathematical modelling studies, particularly with respect to fields near metallic implants, in workers exposed to fields in harsh environmental conditions and at very high frequencies (THz), continue to add to the expanding knowledge database on the characteristics of the complex electromagnetic environment we live in today.

## 1. Introduction

Swinburne University in Melbourne, Australia has had an active research program in tissue interactions of both radiofrequency (RF) radiation and extremely low frequency (ELF) electric and magnetic fields (EMF) since 1989. The research was, to a certain extent, sparked originally by local controversies in relation to power-line and phone base-station siting, but it also grew out of a period during which one of the authors (AW) worked at the U.S. Environmental Protection Agency (US EPA) on related issues [1,2]. After a quarter of a century, the issue of whether or not low-level ELF and RF fields in the environment can be linked to adverse health effects has still not been resolved, despite a worldwide extensive research effort on many levels. Research of possible health effects of these fields, together with their use for therapeutic purposes, has given rise to the name of the discipline, bioelectromagnetics. The society of the same name has sponsored annual conferences since 1978, with the choice of areas of study by the Swinburne group being strongly influenced by the ‘hot topics’ emerging at these conferences. This article will review some of the contributions to the research literature database from this particular group, with some reflections on reasons why identifying unequivocal effects and credible interaction mechanism(s) (relevant to environmental levels) has proved elusive. However, understanding of the patterns of electromagnetic energy absorption has increased dramatically over this same period.

## 2. Radiofrequency Research

RF radiation, if sufficiently intense, is known to cause tissue heating (at lower frequencies it can also cause nerve and muscle stimulation, but the heating, or thermal effect, is the major concern at telecommunications frequencies). The measure of “dose“ is the specific absorption rate (SAR), which represents the rate of absorption of energy per unit mass (of tissue) and is directly related to the in situ rate of rise of temperature. However, biological effects have been reported to occur at levels below which an appreciable temperature rise would be expected. Since there are obvious implications for safety standards if these effects could be substantiated (and shown to have consequences for health) a considerable effort has gone into researching these so-called “non-thermal” effects. 

### 2.1. Cellular Studies

**Figure 1:**
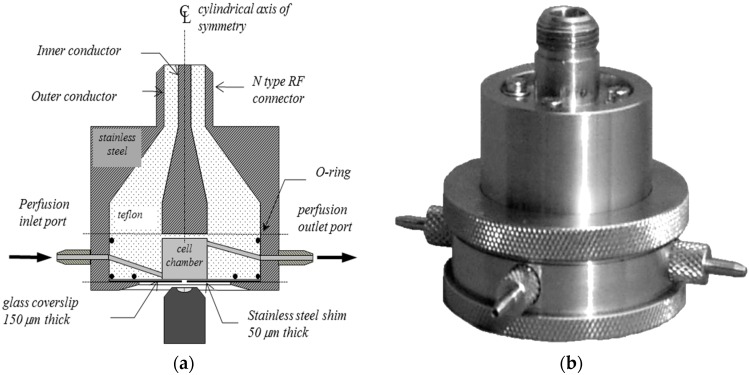
The RF chamber consisting of a cell medium chamber at the end of a modified coaxial transmission line. It is designed to fit on any inverted microscope stage and is used with standard microscope objectives. A glass coverslip forms the floor of the cell chamber and the biological material is viewed through a central 1 mm diameter hole in an underlying steel shim via laser scanning confocal microscopy. The shim shields the cells from RF reflections from the objective. The chamber is shown in vertical cross-section in (**a**); and in perspective view in (**b**). Normally, a coaxial cable from the RF signal generator is attached at the top and the side ports are used to allow perfusion or temperature measurement via fluor-optic probes.

One such “non-thermal” effect, reported in the late 1970s. is the so-called “calcium effect” in the avian brain [3], which appeared to manifest itself as increased (or in some cases decreased) efflux of Ca^++^ from brain hemispheres when exposed to amplitude-modulated RF. This effect seemed to be rather specific in conditions required to observe it (power and frequency “windows” [4], such that if the parameters were either too high or too low the effect was not observed). Our early studies were guided by a push to clarify these putative phenomena, particularly because of the U.S. EPA connection [4]. We attempted to replicate work on calcium levels in the amphibian heart [5], using ^45^Ca as a tracer (all experimentation involving animals was approved prior to commencement by the Swinburne University Animal Ethics Committee — see “Acknowledgements” section for full list of approval codes). We were unable to identify any unambiguous significant effects of 240 MHz exposure (16 Hz modulation) at SAR values ranging from 0.15 to 0.36 mW/kg [6], a range encompassing the value of 0.3 mW/kg showing significant increased Ca^++^ efflux in [5]. At one of the SAR values chosen (0.18 mW/kg) a reduction (*p* < 0.05) was observed, but only in comparison to pooled controls from three runs of experiments. It was considered that because of the multiple comparisons, this isolated result could not be used to support the notion of a calcium effect. Due to the high degree of variability between samples when using the radioactive tracer method, it was decided to move to a more sensitive method of estimating cell calcium to reduce random variation. We, therefore, adopted the use of calcium-specific fluorescent dyes which, when coupled with confocal microscopy, allowed the monitoring of intracellular calcium in real-time. In order to compare cells with and without RF exposure, a specialized chamber was constructed (Figure 1). The cells were maintained in a perfused monolayer culture within a cavity where the electric (E) and magnetic (H) fields were determined by modelling. A small (1 mm) hole at the entrance to this cavity allowed laser scanning images to be formed without perturbing the SAR values in the monolayer. A cell line of immortalized T-lymphocytes (Jurkat cells) were used for this experiment [7]. Exposure within the cell monolayer was arranged to be at the local maximum limits for the general public groups (2 W/kg, with both continuous wave and GSM pulsed 912 MHz RF). Comparisons were between periods of RF exposure and, in general, Ca^2+^ levels were unaffected by RF exposure, but there was a change at the *p* = 0.03 level in the spectral content of the variations in Ca^2+^ levels for one particular exposure condition (for activated cells exposed to pulsed waves). Since we were testing a number of endpoints, the critical *p* value needs to be lower than 0.05 to consider this change as significant. However, it was flagged as a finding of interest for further study.

A recurring issue in in vitro work is the assessment of a possible unintended rise in temperature, manifesting biological effects which appear to be via a non-thermal mechanism but are more likely to be due to localized heating. We have developed probes which allow for the direct measurement of change in temperature, due to the changes in fluorescence in cytoplasmic dyes. Typically, these changes are of the order of a few % per degree rise, enough to be able to track changes in real-time with resolutions below 1 s. The use of confocal imaging allows three-dimensional reconstruction of temperature profiles with a spatial resolution of a few μm. We have used the exposure chamber shown in Figure 1 to follow changes of temperature in RF exposed tissues [8], including fresh brain slices [9].

A recurring theme in bioelectromagnetics safety research has been the question of whether the hormone melatonin confers some protection against genetic damage which. in the case of RF. had been posited in several studies (for example, [10,11]**,** and see Section 3.1 below). In an experiment on blood samples from healthy volunteers [12], the rate of micronuclei formation was compared between modulated and unmodulated 2.45 GHz radiation, with and without melatonin (2 mM). There were no significant differences due to RF exposure, but in a positive control, using 1.5 Gy of ionizing radiation, where there was a 10-fold increase in micronuclei incidence, the effect of melatonin was to reduce this increase by 35%.

The bone marrow, since it is the most proliferative tissue in the body, was considered to be a sensitive site for testing for RF effects. In several series of experiments long bone explants from rats were exposed to 900 and 1800 MHz radiation, both continuous wave and pulse-modulated, and at two levels of SAR (2 and 10 W/kg, approximately) [13,14]. Both proliferation rate and genotoxicity (via the alkaline comet assay) were measured, but RF radiation did not produce any significant alterations, when compared to sham.

In all of these studies, modelling software has been used to estimate SAR and in many cases temperature changes in these isolated tissue samples during exposure to RF. Further details of this approach will be described in Section 2.3 below.

### 2.2. Human Volunteer Studies

The main driver in these studies was concern that cognitive function, in particularly memory and higher executive functions could be affected by emissions from mobile phone handsets (see, for example, [15]). In our first project, human volunteers were exposed to radiation similar to that received during a mobile phone call (all experimentation involving human volunteers was approved prior to commencement by the Swinburne University Human Research Ethics Committee — see “Acknowledgements” section for list of approval codes). Specially adapted mobile phone handsets were used. These were characterized in terms of RF energy output by in-house testing. In the first set of experiments, 120 participants were given a battery of neuropsychological tests [16]. In some of the eight tests changes with *p* < 0.05 were obtained. Of these, simple and choice reaction times showed strong evidence of impairment but performance on the trail-making task improved, supporting the hypothesis that RF emissions improve the speed of processing of information held in working memory. However, the number of comparisons makes drawing firm conclusions difficult. A review of the literature at the time highlighted the relative consistency in RF effects on the so-called “alpha” band of the EEG signal [17], so these findings helped formulate specific hypotheses for the next studies.

In the next series of studies, the ability of a further 120 participants to respond to visual and auditory stimuli (with and without RF exposure) was assessed by measuring brain electrical activity. The quality of their sleep during the subsequent night was measured. This second part also measured the products of melatonin metabolism in overnight urine (since melatonin is an essential marker of sleep and circadian rhythms, as discussed in Section 3.1 below). Before the main study was commenced, a pilot study of 12 volunteers was undertaken. In this, multiple measures of response to an auditory stimulus were altered, but no definitive conclusions could be drawn given the small sample size [18]. However, the findings were used in forming specific hypotheses for the full study.

Testing of these 120 human subjects for the full event-related potential (ERP) study took place in 2002–2004. The principal results are presented in two reports: in the first [19] it was concluded that there was no significant difference between exposure conditions for any auditory or visual electrical activity or reaction time. The second report (a further analysis of the data by the first author [20]), concluded that although there was no overall change to the chances of a statistically significant effect occurring in the period following exposure cessation, there was a reduced chance of such an effect on the side opposite to the exposure source during this period. A further study at Swinburne [21], comparing 2G and 3G exposure in adolescent, young adult, and elderly groups of volunteers showed that the increased alpha power was only significantly increased in the 2G young adult group.

For the sleep variables and urinary melatonin component of the study, 60 subjects were recruited [22,23]. The principal findings were that mobile phone exposure prior to sleep may (a) promote rapid eye movement (REM) sleep; and (b) modify brain activity in the first non-rapid eye movement sleep period. Total night-time melatonin output was unchanged, but there could be an effect on melatonin onset time.

The principal way of testing was to measure brain electrical activity via electrodes attached to the scalp. Although in the case of the sleep study exposure occurred before the electrodes were applied, the ERP study had exposure coinciding with measurement. We, therefore, needed to ensure that the RF signal was not being picked up and demodulated by the electrode montage or the amplifier front end and, moreover, that the RF was not contaminating the auditory or visual stimuli, which would give clues on whether or not RF exposure was occurring. By using a “phantom scalp” constructed of material with electrical properties similar to a real scalp, the effects of pickup were determined and found not to be an issue [24]. A related question, whether or not the use of stick-on electrodes altered the SAR value within brain tissue was also investigated, via modelling [25]. It was found that SAR was actually reduced, which argued against any identified electrophysiological changes being thermal in origin.

The majority of this work transferred to the University of Wollongong around 2005 (see the article by Loughran et al. in this issue).

### 2.3. RF Modelling Studies

As mentioned, the experimental evidence for a “calcium effect”, with frequency and amplitude “windows” presented a challenge—could these “windows” be predicted from a mechanistic point of view? In the model of Thompson et al. [26,27], non-linear cooperativity was incorporated into a lattice model of Ca^2+^ receptors in which the applied modulated RF field could induce a conformational change. Although there were no direct estimates of whether the conformational energies required were actually available in the RF at the levels reported (10–100 W/m^2^) the model was successful in reproducing the amplitude windows reported by Blackman et al. [4].

Direct analytical modelling was also applied to the question of field distributions close to the feed-point of antennae. In this region, the fields set up by moving charges within the antenna wires have a reactive influence, making their prediction difficult. Since this is the situation when using a phone handset, this is an important area to study. Kurniawan [28,29] used closed-form solutions of Maxwell’s equations (with some heuristic adjustments) to predict fields both in the air space between the antenna and the skin and within specific tissues within the head. This work gave an explanation of some discordant results which have appeared in the literature and also presented easy-to-use computational tools for evaluating fields in near-field situations.

The chamber shown in Figure 1, which was used in the in vitro work already described was characterized by the use of numerical software [30]. The use of a “zoom” feature in the solver used (FEKO) was particularly useful in plotting the field and Poynting vectors in the region directly above the 1 mm viewing hole where the adherent cells would be situated. This modelling revealed the distribution of SAR values in the viewing region, to accurately predict the relevant values within cells or tissues, both in the experiments where the temperature was held constant, by the use of perfused media, or in the dye-derived temperature measurement studies, where by ceasing perfusion, the temperature was allowed to rise.

#### 2.3.1. Mouse Models

Anatomically detailed computational models of mice—a female, a pregnant female, a male, and a foetus—were developed [31] to provide dosimetry support to scientific studies. Six Swinburne students, co-authors of the paper, spent hundreds of hours meticulously assigning 49 tissues to the finite-difference mesh of the mice. A key outcome was that the model of the pregnant female mouse was used to accurately characterize the SAR and levels and resultant temperature change in the foetuses in studies at the Institute of Medical and Veterinary Science (IMVS) in Australia [32,33,34]. In a computational analysis the pregnant mouse model was subject to RF exposure as would be experienced in the IMVS studies, where a radial cavity exposure system was operated at a frequency of 900 MHz. It was found that the SAR levels in the foetuses were around 14% lower than the average values of the mother and the peak temperature increase was around 45% lower than the values in the mother. All mouse models are freely available to be used by other researchers.

#### 2.3.2. Comprehensive Tissue Thermal Parameters Database

Accurate computation of the thermal effects of RF exposure in human and animals requires accurate input values of the tissue properties: specific heat capacity (*c*), thermal conductivity (*k*), blood perfusion rates (*m*), metabolic heat production (*A*_0_), density (*ρ*), and water content (*w*). In [35,36] we documented over 150 key papers and books that included measurement data for those six properties and formed a summary table of the data to provide a valuable resource to researchers. The table provided data for 43 tissues and the average and range of the thermal properties was provided. This resource is available to the biological thermal modelling community to help improve the accuracy and consistency of published results.

#### 2.3.3. Thermal Regulation in Workers Exposed to RF in Harsh Climates

A study performed in conjunction with IBM Research, using the computational facilities of the Victorian Life Sciences Computation Initiative (VLSCI), built a sophisticated computational environment that is able to determine a realistic thermal profile of an RF worker wearing protective clothing and subject to adverse environmental conditions, including high humidity and high ambient temperature [37]. The calculations in the paper used a finely discretised, heterogeneous human body model, subject to the maximum allowable reference level for a 1 GHz RF electromagnetic field for a worker, and also subject to the adverse environmental conditions just mentioned. The model also incorporated physiological control mechanisms for sweating, shivering, and vasoconstriction/dilatation, together with two layers of appropriate clothing. An observation was that while electromagnetic fields at the occupational safety limit will contribute an additional thermal load to the tissues and, subsequently, cause an elevated temperature, the magnitude of this effect is far outweighed by the conditions including the ambient temperature, relative humidity, and the type of clothing worn. Ongoing studies are examining a range of conditions for an RF worker and the aim is to input into the setting of exposure limits so that a wide range of environmental scenarios in which an RF worker may find themselves are covered accurately by the standards.

#### 2.3.4. Terahertz Radiation Absorption by Biological Material

What used to be termed the “Terahertz Gap” (frequencies between millimetre waves and far-infra-red) are now being increasingly researched for telecommunications, agriculture, military, and medical applications. Since this form of radiation is strongly absorbed, study of the electrical properties of skin and small organisms is increasingly relevant. Recent work from the group is studying the absorption properties of cell components in order to adequately model the deposition of energy from this form of radiation [38].

### 2.4. RF Dosimetry Studies

Much effort has focused on determining the appropriate metrics for assessing localized RF exposure over the frequency range 1–10 GHz [39,40,41]. Our findings have been used by international standards-setting bodies and regulators to clarify whether the current metrics are appropriate or whether they should be modified. When setting protective limits for localized tissue heating, the SAR at a particular point is mass averaged in recognition of the thermal diffusion properties of tissues. In our studies we sought to determine the optimal averaging mass, noting that different tissues diffuse heat at slightly different rates. Furthermore, over the frequency range 1–10 GHz the penetration depth into the human body of an electromagnetic field drops off from an order of magnitude of several centimetres at 1 GHz down to just a few millimetres at 10 GHz. Thus, we further sought to determine the cutover frequency at which SAR should be replaced as the exposure metric by the incident power flux density, *S_inc_*, a measure of the field that is incident on the surface of the body. Our computational analyses consisted of both sophisticated human body models and two-dimensional planar representations of tissue slices. The latter allowed a greater number of scenarios to be considered through reduced computer solution time. Our studies recommended that SAR should be used for 1–6 GHz, averaged over a 10 g mass, and *S_inc_* used for 6–10 GHz.

In [37] we proposed an alternative metric, the volumetric energy absorption rate (VAR), which is similar to SAR except that tissue density is not included in the formulation. VAR was found to be a slightly better correlate to tissue temperature change than SAR. There is no reason per se as to why density should be included in the formulation of a metric for RF energy absorption. Including density can lead to misleading values, best noted by the example of bone tissue. The density of bone is around twice that of many tissues and, as a result, the SAR levels in bone may be around half that of neighbouring tissues for similar levels of absorbed energy.

There are many people who carry metallic items inside their bodies, including orthopaedic plates, screws, and wires, and electronic devices, such as pacemakers. Whenever an RF field impinges on an implant, the field is scattered and the energy of the incident field may be redistributed to produce significant peak SAR concentrations around certain parts of the implant. In one of our computational studies [42] an adult male, representing an RF worker, was modelled to be standing on a conductive ground plane and exposed to a 40 MHz vertically-polarized plane wave field, close to whole-body resonance where maximal induced current flows are expected in the legs. Over a series of computations, metal plates of varying lengths (50–300 mm long) were attached to the tibia in the left leg. As well as the need to satisfy SAR limits, it was appropriate in this study to consider whether the exposure satisfied limb current reference levels. It was found that significant increases in limb current, SAR, and temperature increase values occur when a tibial plate is present and that the values increase with increasing length of the plate. It was recommended that in order to satisfy the limb current levels, measurements should be performed on all RF workers when high exposure levels are expected. If due caution is taken to satisfy the incident field and limb current reference levels then the peak 10 g SAR values will also be below recommended limits.

In addition to the work just described, the group has been involved in international efforts to develop measurement methodologies for new and emerging technologies [43] and to minimize electromagnetic compatibility issues in hospitals [44]. The group’s work has been assisted by the anechoic chamber facilities at Swinburne for both testing of measurement equipment and the quality assurance of biological exposure setups. 

## 3. Extremely Low Frequency Studies

ELF fields at sufficiently high magnitudes are known to cause acute nerve and muscle stimulation. Concerns that low-level ELF (particularly magnetic field) exposure could lead to harm arose following the release of the epidemiological study in Denver in 1979 which indicated a link between high current configuration street wiring and childhood cancer [45]. In order to determine whether magnetic fields normally encountered in the environment could lead to subtle physiological changes which could possibly linked to cancer development or progression we decided to concentrate on controlled studies within the laboratory involving human volunteers. 

### 3.1. Human Volunteer Studies

**Figure 2:**
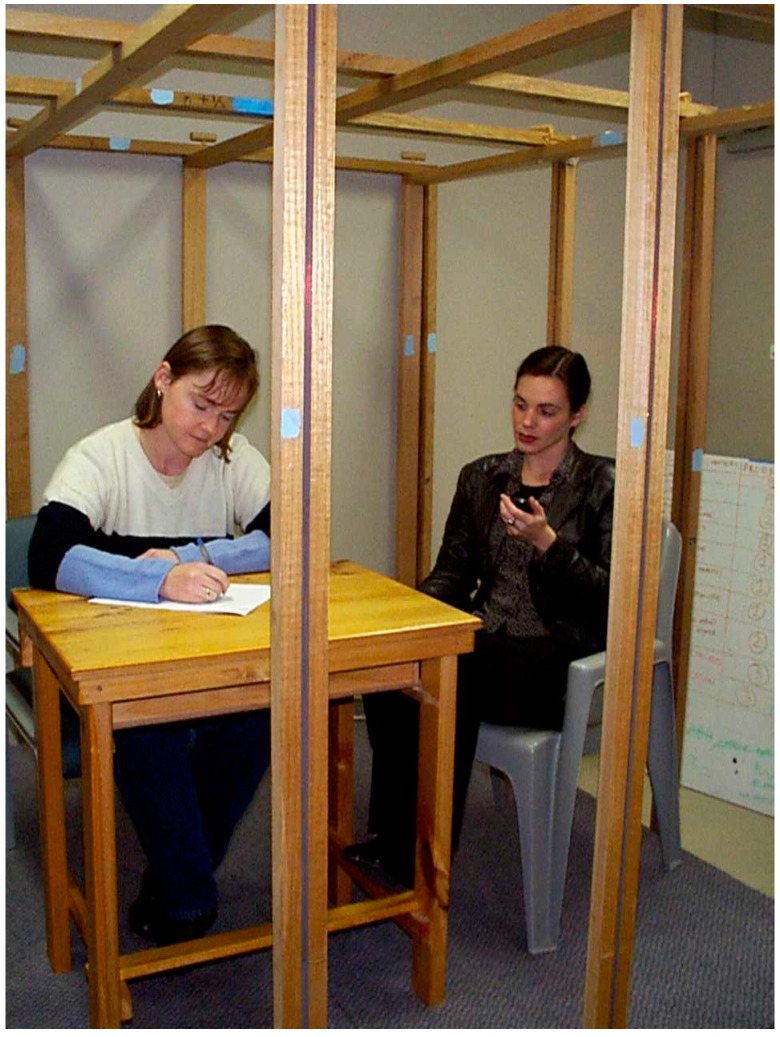
The ELF exposure system consisting of orthogonal square Helmholtz coils with spit windings—this allows current to flow both during sham (cancelling mode) and field (augmenting mode) to avoid audible cues due to the 50 Hz “hum”. The two sets of coils are 90° out-of-phase, giving rise to circularly-polarized fields, similar to those near power transmission lines. All items within the coils are made from non-ferrous materials. The central region with 0.8 m radius has a field uniformity within 2%.

These studies were instigated in response to interest in the so-called “melatonin hypothesis” [46], which is the notion that attenuated melatonin rhythms due to ELF exposure reduces free radical scavenging by melatonin and might thereby increase the risk of cancer. A cohort of 30 adult males were exposed to 50 Hz circularly-polarized magnetic (*B*) fields, magnitude 20 μT, (28 μT resultant) in a large set of orthogonal Helmholtz coils [47]. This is shown in Figure 2. Each participant attended at least three times, the first to determine the exact melatonin onset time in the evening (for each individual, when this occurs is remarkably constant) and then another two times when they received a 2 h exposure or a sham exposure in a double-blind and counterbalanced design. There were three regimes: exposure pre-, during, and post-melatonin rise. Those receiving exposure pre-rise showed a significant delay of around 30 min on melatonin onset. Analysis of change of onset time between screening and sham night showed a Gaussian distribution, as expected, but the change in onset time between sham and exposure nights showed a distinct bimodal distribution, suggesting that around 20% of participants were ‘responders’ and the remainder “non-responders”. A small number of participants (identified in the first round of testing as responders) returned for further testing. There was some consistency in repeat behaviour and one individual showed consistent delays of around 1 h on five pairs of test nights. In order to determine whether the rate of change of magnetic field with time (usually denoted *dB*/*dt)* rather than magnetic field itself (*B)* might be the field quality relating to the (putative) effect, a small additional study was performed, comparing square (large *dB*/*dt*) versus sinusoidal (smaller *dB*/*dt*) fields. Both types of field produced average delays of similar amounts, but the square wave group was associated with significant reductions in the amount of melatonin released during the night. The study was not without its shortcomings, principally the need to re-categorize the exposure periods a posteriori (but blinded with respect to exposure condition), but also the unbalanced numbers in the three categories due to irregular attendance of some of the participants. It was unfortunate that this preliminary study (which, at the time, was the largest in terms of the cohort size) could not have been followed up through further funding.

Additionally, around this time there was considerable interest in whether heart rhythm could be affected by ELF. The same exposure system (Figure 2) was used to study heart rate (HR) and heart rate variability (HRV) in several series of experiments on adult volunteers. Following a pilot study, which showed an approximate 5% (transient) slowing of HR when the field was switched on, a follow-up study confirmed this, but with a smaller (2%) amount of slowing [48]. This study also examined HRV, which showed a lowering of low band (0.02–0.15 Hz) to high band (0.16–1.0 Hz) ratio for the field on compared to field off, for both on-to-off and off-to-on switching. To avoid contamination of the low band region by slow breathing patterns the respiratory rate was controlled, as is the common practice [49]. Participants breathed according to the following three patterns: spontaneous; in time to a 0.2 electronic metronome; and breathing against a closed glottis (Valsalva maneuver) at 15 s intervals, to coincide with the field switching. The controlled breathing was particularly effective in eliciting significant field switching-related changes in low band power reduction. High band power also significantly increased for on-to-off switching, but not for the other direction. Low band power reduction is compatible with increased vagal activity, so a further series of experiments were conducted utilizing a tilt table, to provoke a reduction in vagal activity. Here participants, under controlled breathing conditions, were initially supine, but were tilted to head-up 60°, with a comparison of changes in HRV obtained with real fields compared to sham. Although the anticipated changes in low and high band power occurred with tilting, there were no significant differences between real and sham field conditions [50]. Another possibility which was pursued was whether shielding the head from the field would distinguish between effects directly on the heart and effects involving the brainstem and baroreceptor regions. In these experiments, no field-related changes in low band power were found [51]. This experiment involved two sham periods in addition to the field period, indicating the difficulty of replicating subtle effects, if indeed they only occur under highly specific conditions, if they occur at all.

The third avenue of these volunteer experiments was to investigate effects of these fields on cognition and EEG. In the case of the former, a series of verbal and written tests were conducted with a comparison of performance during periods of field compared to periods of sham, with appropriate counterbalancing of order and allowance for practice effects. Most types of tests showed no differences, but two (verbal recall after interference and trail-making task) showed significant impairment in the “field on” condition [52]. However, the number of participants (30) was probably insufficient to give definitive results. The measurement of EEG responses is a greater challenge, because of the direct pickup from the external fields. By a combination of axial lead routing, shielding, notch analog and digital filtering, the evoked responses appeared uncontaminated by 50 Hz [53]. Visual and auditory evoked potential latencies were measured in a group of 40 participants, but no changes in these latencies were noted. In the other 27 written and verbal tests conducted in this series of experiments choice reaction time accuracy, word recognition accuracy and numerical working memory showed impairments at the 5% level, but no adjustments were made for multiple comparisons, so no definitive conclusions could be drawn [54]. Again, noting that all changes were detriments, but that in different series different end-points were affected, suggests that in order to arrive at definitive conclusions, sample sizes in the hundreds are necessary.

### 3.2. ELF Modelling Studies

Electrical power line maintenance workers routinely work on energized lines (live line work). Here, workers are exposed to high magnetic fields, but because they are at the same potential as the line, the electric field exposure is much smaller and mainly occurs during the transition from the tower superstructure to the line via an insulated ladder. A modelling study [55] considered the perhaps unlikely scenario of a pregnant female worker in this role and the induced currents in the foetus. In a somewhat simplified model, it was shown that high current densities were likely in the amniotic fluid and the placenta and the current density in the foetus exceeded the (then) public limit in the international standard, especially if the line is parallel to the long axis of the mother’s body. In the time since the publication of this paper, more highly-structured models have been developed for mother and foetus, but the conclusions still hold.

## 4. Standards Development

The Australian national radiation laboratory, ARPANSA, has had strong links with the Swinburne Bioelectromagnetics Group, particularly in the development of RF and ELF Standard documents. Two members of the group were part of the Working Group for the 2002 RF Standard and one of the authors (AW) was also involved in a recent review of this standard [56]. This standard, which has the same limit values as the 1998 standard of the International Commission for Non-ionizing Radiation (ICNIRP) [57], provides additional reviews and resources and refers to “precautionary” advice, that is, the minimization of inessential RF exposure. It was one of the first standards to do so. The review concluded that the basic restrictions in the standard were still adequate, but that the safety margins at some frequencies might be less than earlier thought. In relation to ELF, AW chaired the working group, publishing a discussion paper of the rationale underlying basic restrictions [58] and discussing possible scenarios for local standards [59]. Harmonization of local standards with international standards has always been an important consideration, so although the draft local standard had a number of innovative features, including the first to incorporate advanced computer modelling, on the publication of the ICNIRP ELF standard in 2010 [60], this standard took precedence. As a member of the Scientific Expert Group of ICNIRP, AW gives input into international standards development for both ELF and RF.

## 5. Discussion

The work of this group has been of two types: health effects and dosimetry research. The abiding feature of health effects work from this group, which is also observed in other, similar, groups, is the lack of consistency of results. It is to be emphasized that the exposures used in these experiments have been at or below the level set in international standards so, therefore, no deleterious effects would be expected and the reason for carrying out these experiments is to ascertain whether any reduction in levels is indicated. The overall conclusions that we come to from our experiments is that the exposure limits given in the relevant standards (for example, those issued by ICNIRP) are, in the main, adequate. However, some of the dosimetry studies (such as some we have been involved in) indicate that ongoing review is necessary. Absence of evidence is not evidence for absence, and the ubiquity of exposure near these levels requires on-going surveillance. The International Agency for Research on Cancer (IARC) categorization of both ELF magnetic fields and RF as possibly carcinogenic to humans implies that there is still uncertainty. It is useful to reflect on why some of the experiments reviewed here have yielded significant field-related changes and why (in the case of the heart rate experiments referred to above, for example) significant changes noted in pilot studies have not been repeated in follow-up studies. Certainly, the use of larger sample sizes increases precision, but also studies subsequent to the pilot often include better control of conditions and reduction in measurement errors. There has also been some recent discussion [61] on the question of whether a *p* value of less than 0.05 (or 0.05/*n* if *n* is the number of comparisons) is truly an identification of an “effect”. Certainly, there needs to be careful generation of specific hypotheses to test. Although these can be generated from pilot experiments, some reference to a putative mechanism is important: here there are no credible mechanisms, so the possibility of “*p* hacking” (that is, to keep making comparisons until changes at the *p* < 0.05 level are uncovered) is very present. As has been pointed out [61], this is especially prevalent where there is a quest to “chase small effects hidden in noisy data”. Effect size is defined as the treatment group mean minus the control group mean divided by the control group standard deviation and is suggested as a more useful parameter to report in this regard. Lack of reproducibility in science is in fact quite common. A recent survey of research scientists [62] has revealed that, in biology, 60% of respondents failed to reproduce their own experiments and 77% had failed to reproduce results reported by others. The top three reasons cited for these failures are: selective reporting; pressure to publish and low statistical power, or poor analysis. Although not always followed, we have sought to follow best practice: the use of a double-blind counterbalanced design; the careful characterization of exposure parameters; the use of pilot data to generate specific hypotheses for larger studies and, in some cases, the reporting of effect sizes rather than *p* values. Often, we have asked others within the laboratory to repeat our earlier work to improve quality control. Over a 25-year period laboratory techniques have improved significantly in precision and in the level of quality control. In this regard, if there were genuine low-level effects occurring, then improved precision would be expected to yield more definitive evidence, but this has not been our experience. It is difficult to speculate on the reasons such differences in outcomes reported by different research groups, although differences in precise experimental designs or exposure parameters may play a part. In a review of putative low-level ELF mechanisms by one of us nearly 25 years ago [63] “the problem of coming to a consensus view in an area where experimental findings lack robustness and theoretical frameworks lack coherence” was highlighted. This has not really changed. The interaction energy in weak (environmental) fields is so low that some form of biological amplification is needed for the reported effects to be credible. This biological amplification mechanism has not been identified. Although human volunteer experiments are, perhaps, more realistic in terms of investigating an exposure which may affect multiple interacting systems within the body, in vivo/in vitro experiments offer much greater control over both exposure and ambient conditions, which would be expected to lead to greater reproducibility. However, in our experience this has not been the case.

Dosimetry research, similarly, has seen a dramatic increase in precision over 25 years. At the start of this period, models of the human body were homogeneous spheroids or gross segments. Body models now have less than 1 mm cube resolution with 30–40 different tissue types identified from MRI and other imagery. Each of these tissue types has well-characterized electrical and thermal properties. At the same time, knowledge of the characteristics of the fields we are exposed to (in occupational and residential/communal settings) have been vastly improved, through mathematical modelling and extensive surveys. As communication systems continue to exploit higher frequencies (because of higher data capacity) interaction with skin and small organisms continue to be a major focus of research, which is certainly the focus of some of our research. 

## 6. Conclusions 

Over its 25-year history the Bioelectromagnetics Group has received support from national competitive grants and from industry research support schemes. It has been a node for both the Australian Centre for Radiofrequency Bioeffects Research (ACRBR) and the Australian Centre for Electromagnetic Bioeffects Research (ACEBR—see article in this edition). It has benefitted from industry collaboration and with national regulatory authorities. The focus has been (a) to identify bioeffects relevant to human health at levels below the present standards; and (b) to improve the estimation of induced currents and fields within biological material, including the human body. Regarding the former, although in pilot experiments some significant field-related effects were observed, follow-up experiments (in the main) failed to reproduce the initial findings. Our conclusions are that for RF exposure below the exposure limits, we have not demonstrated any effects on cell Ca^++^ levels or genotoxicity. In human volunteer experiments, some experiments were suggestive of subtle changes in EEG, but not in melatonin output. For ELF exposure in human volunteers, there was weak evidence of changes in melatonin onset time, but no definitive changes in HRV nor cognition. Regarding dosimetry research, the studies of fields around implants, in workers in harsh environments, and at THz frequencies is on-going and represents, we believe, a useful contribution to knowledge. In relation to the general question of whether the current standards (derived from studies of short-term effects) are adequate for the long term or ignore particular low level effects (regarded by some as having been established), there is still more work to be done. ICNIRP, via its Scientific Expert Group, is continuing to examine these possibilities.
